# Assessing the Potential for Interaction in Insecticidal Activity Between MON 87751 × MON 87701 Produced by Conventional Breeding

**DOI:** 10.1093/ee/nvz082

**Published:** 2019-07-01

**Authors:** Steven L Levine, Jennifer M Fridley, Joshua P Uffman

**Affiliations:** Regulatory Sciences, Bayer Crop Science, Chesterfield, MO

**Keywords:** MON 87751, MON 87701, additivity, concentration addition, response addition

## Abstract

Pyramiding (combining) of plant incorporated protectants (PIPs) with insecticidal activity in genetically engineered crops is a strategy used to improve efficacy as well as delay potential resistance for a specific group of targets. In some countries, a regulatory risk assessment is required for breeding “stacks” expressing multiple PIPs and these countries may require an assessment of potential interaction among the PIPs. This study evaluated whether combining soybean events MON 87551 and MON 87701 results in a toxicological interaction that effects a species that is controlled by each event. MON 87751 coexpresses the Cry1A.105 and Cry2Ab2 proteins and MON 87701 expresses the Cry1Ac protein. EC_50_ values for MON 87751 and MON 87701 were comparable in diet-incorporation bioassays using corn earworm (Lepidoptera: Noctuidae, *Helicoverpa zea*) and the observed combined activity of the stack was consistent with predictions of additivity (i.e., no interaction). Under the concentration and response addition models, predicted and observed median effect levels differed by <10%. These results demonstrate independent action at the median effect level between the insecticidal activity of MON 87751 and MON 87701. Taken together, no interaction between these PIPs and acceptable margins of safety for the individual proteins to nontarget organisms, it is appropriate to bridge back to the risk assessments for the individual products that demonstrated environmental safety of stack products containing both MON 87751 and MON 87701.

Genetically engineered (GE) crops producing plant incorporated protectants (PIPs) for control of economically important pests have been commercially cultivated for almost 25 yr (ISAAA 2018). First-generation GE crops only produced a single PIP for insect control. However, to improve the efficacy, pest spectrum, and delay potential resistance, PIPs with different modes of action are now commonly combined to target a specific group (e.g., pyramided for Lepidoptera control) to have efficacy against different orders (e.g., Lepidoptera and Coleoptera; Head and Greenplate 2012). GE crops with combined insecticidal PIPs and or herbicide tolerance traits (i.e., stacked trait products) produced through conventional breeding are now commercially available for cotton, corn, and soybean (ISAAA 2018). Recently, Monsanto Company developed and registered a stacked trait soybean product to control targeted pests that combined events MON 87751 and MON 87701 through conventional breeding (USEPA 2016, ISAAA 2018). MON 87751 was produced by stable insertion of the coding sequences of the Cry1A.105 and Cry2Ab2 insecticidal proteins. MON 87701 was produced by stable insertion of the coding sequence for the Cry1Ac insecticidal protein (USEPA 2010). Both MON 87751 and MON 87701 provide protection from feeding damage by several lepidopteran pests. In addition, MON 87751 × MON 87701 has been conventionally bred with Roundup Ready 2 Xtend (MON 87708 × MON 89788), resulting in a stack comprised of MON 87751 × MON 87701 × MON 87708 × MON 89788. MON 87708 is a dicamba-tolerant soybean that produces a dicamba mono-oxygenase protein from *Stenotrophomonas maltophilia* to confer tolerance to dicamba (3,6-dichloro-2-methoxybenzoic acid) herbicide and MON 89788 soybean confers tolerance to glyphosate by expressing the 5-enolpyruvyl-shikimate-3-phosphate synthase protein (EPSPS).

In 2009, the United States Environmental Protection Agency (USEPA) suggested data requirements for stacked PIPs produced by conventional breeding of previously registered events (USEPA 2009a,b). The USEPA’s evaluation of breeding stacks utilizes data from existing registrations of each parental (single) line in conjunction with well-defined bridging studies. The purpose of one of these bridging studies is to demonstrate that the combined activity of the PIPs in the stack does not affect existing risk assessments for any of the previously registered single events that comprise the stack. The EPA refers to this study as the “synergy” study and it has this title to show the interest in evaluating the potential for supra-additive activity of combined PIPs and implication for the environmental risk assessment (USEPA 2009a). The term synergy has many definitions and carries negative connotations in some fields. Therefore, the term “greater than additive” (GTA), which is more precise and objective, will be used here instead of “synergy.” In the toxicological literature, a GTA interaction occurs when the combined effects of two components are significantly greater than the sum of the effects of each component given alone (e.g., 2 + 2 = 20; Casarett et al. 2003). Data that are consistent with additivity (e.g., 2 + 2 = ~4) do not represent an interaction and are commonly termed zero-interaction or no-interaction (Könemann and Pieters 1996).

Test species for interaction studies should be susceptible to one or more of the PIPs and be amenable to a laboratory bioassay. For practical reasons, interaction studies for PIPs are usually carried out with target pest species and it is their sensitivity to one or more of the PIPs, not their pest status, that is important. The rationale for testing a sensitive species is that increases in toxicity are more likely to be detected. If GTA interaction is not demonstrated against a susceptible species, and there are sufficient margins of exposure for nontarget organisms (NTOs) for the individual PIPs, then no adverse effects to NTOs would be expected from combining two or more PIPs (USEPA 2009a). In other words, if the PIPs in a combined-trait product show no-interaction with a susceptible species, there are adequate margins of safety for the individual components, and there is comparable environmental exposure for the single and the stack (i.e., comparable expression levels in both), then the safety assessment for the stack can be bridged back to the existing studies that support the safety assessment for the single products. For the Cry1A.105, Cry2Ab2, and Cry1Ac proteins, there is highly comparable protein expression levels in the single and the stacks (CTNBIO 2018). This approach of independent assessment has a long-standing application in the field of toxicology and has been referred to as the principal of independent assessment. (USEPA 2004, Levine et al. 2016). The Federal Insecticide, Fungicide, and Rodenticide Act Scientific Advisory Panel (FIFRA SAP) recommended to USEPA that for combinations of PIPs that have been previously registered as individual events and have a proven safety record, that GTA interaction less than 10-fold should not trigger additional NTO testing (USEPA 2009b). The rationale for this recommendation provides a risk-based approach to address GTA effects in the context of an environmental risk assessment. In addition, this approach largely reflects the need to achieve a margin of exposure of ≥10 times the expected environmental concentration, which is the margin of exposure generally required for Tier I NTO testing and assessments (USEPA 2007, USEPA 2010). As an alternative to conducting interaction studies, NTOs can be tested with all PIPs in combination.

This study evaluated whether combining MON 87751 and MON 87701 through conventional breeding results in GTA interaction using the corn earworm, (Lepidoptera: Noctuidae, *Helicoverpa zea*). There are two well-accepted approaches to assess additivity of two or more components and these models are the concentration and response addition models (Finney 1971). Response addition has historically been used to assess endpoints for mixtures with different (i.e., independent) modes of action (USEPA 1986, Green et al. 1995). For this reason, the response addition model has been known as the “independent action” model and the combined response simply equals the sum of the two fractional responses minus their product (Bliss 1939). Therefore, under the principles of the response addition model, components present at doses or concentrations below their no-effect levels (i.e., levels below a toxic threshold) will not contribute to a joint effect, and combined effects from the mixture are not predicted (i.e., 0 + 0 = 0; Könemann and Pieters 1996, Borgert 2004, Price 2010, Kortenkamp and Altenburger 2012). Accordingly, when assessment endpoints are based on no observed effects levels (NOELs), and exposure levels do not exceed a threshold effect level, a finding of no-interaction can support bridging back to the conclusions of the existing assessments for the individual products. Several published Bt interaction studies have used the response addition model to effectively test the hypothesis of no-interaction (Raybould et al. 2010, Levine et al. 2015, Graser et al. 2017).

Concentration addition is an established model for examining interactions between substances having a similar mode of action such as *Bt* Crystal proteins (Finney 1971). A mode of action is generally viewed as a category of mechanisms that share key features or steps (USEPA 1986). The concentration addition model commonly generates more conservative predictions compared with the response addition model when the dose responses are parallel (Finney 1971). However, predictions from both models are comparable for compounds that have concentration–response curves with logistic slope parameters around 1.5 (Olmstead and Leblanc 2005). For these reasons, concentration addition is the USEPA’s default model for assessment of additivity (USEPA 1986, Backhaus et al. 2010). However, both approaches have been effectively used to assess the potential for a GTA interaction between PIPs and a comparison of the results from the two models is presented for the assessment of potential interaction between MON 87751 and MON 87701 (USEPA 2009c, Levine et al. 2015, USEPA 2015b, Levine et al. 2016, Walters et al. 2018).

## Materials and Methods

### Experimental Design and Test Materials

Lyophilized and ground leaf tissue harvested between V6 and V7 growth stages from MON 87751, MON 87701, and MON 87751 × MON 87701 × MON 87708 × MON 89788 plants was incorporated into an artificial lepidopteran diet over a concentration range determined in range-finding assays. The stack that was tested in bioassays included herbicide-tolerance traits because they were part of the commercial product. The bioassay was infested with newly hatched *H. zea*, which is sensitive to Cry1A.105, Cry2Ab2, and Cry1Ac proteins (USEPA 2015a). This study used lyophilized tissue rather than the purified Bt proteins because it is an efficient way to deliver the Bt proteins and the Bt proteins are at the ratio that they appear in the individual and stacked products. In addition, as part of the registration review process, registrants are required to show that the plant-produced and microbially produced Bt proteins are structurally and functionally equivalent. Sixteen impartially assigned and individually housed larvae per treatment replicate were fed for 7 d. At assay termination, the number of surviving larvae and their combined mass was recorded. Growth inhibition was selected as the response variable because it is a sensitive endpoint and shows a large dynamic range (i.e., a high level of efficacy for growth inhibition could be observed) and was not confounded by mortality. Overall, the assay design, analysis, and interpretation reflect the recommendations for toxicological interaction studies presented by Borgert et al. (2001), Levine and Borgert (2018), and the recommendation made by a USEPA FIFRA SAP (USEPA 2009b). Together, these criteria and recommendations reflect the consensus of the literature on interaction analysis developed over decades of research in pharmacology and toxicology and can be applied to data from biologicals, drugs, pesticides, industrial chemicals, and food additives. Briefly, these criteria require adequate characterization of the concentration–response relationship for the individual components and the combination, testing the components at the appropriate ratio(s), stating a clear no interaction hypothesis, evaluating a biologically relevant endpoint(s), having adequate replication within and between assays and performing an appropriate analysis and interpretation of the data.

Exposure for the singles and the stack followed a fixed-ratio or ray-design. *Helicoverpa zea* larvae were exposed to seven lyophilized leaf tissue diet concentrations with each test and control material assayed concurrently. Assays for each treatment were run concurrently and repeated three times on separate days with separate batches of insects to address and assess interassay variability. Control tissue was from a conventional soybean line with similar background genetics to the singles and the stack. All test and control materials (treatments) were grown in the same genetic background and were all grown together under the same environmental conditions. A summary of the test and control substances is presented in Table 1.

**Table 1.** Summary of treatments for *Bacillus thuringiensis* (*Bt*) proteins produced by MON 87751, MON 87701, MON 87751 × MON 87701 × MON 87708 × MON 89788

Treatment*	Phenotype	Expressed *Bt* Protein
Conventional Control	Conventional	None
MON 87751	Insect protected	Cry1A.105, Cry2Ab2
MON 87701	Insect protected	Cry1Ac
MON 87751 × MON 87701 × MON 87708 × MON 89788	Insect protected, glyphosate and dicamba tolerant	Cry1A.105, Cry2Ab2, Cry1Ac

* All test and control materials (treatments) have the same genetic background and were all grown together under the same environmental conditions.

### Tissue Growth and Preparation for *H. zea* Bioassays

Leaf tissue was harvested from plants grown in a growth chamber. Each plant was verified by event-specific Polymerase Chain Reaction (PCR) assay prior to collection as confirmation of identity. Any plants that did not test as expected were disposed prior to sampling. Leaf samples were lyophilized in a VirTis 24 × 48 GPFD Freeze Dryer (SP Industries, Stone Ridge, NY). After one cycle (approximately 2 d), all tissue samples were removed from the freeze dryer to obtain a baseline dry weight in the container. This process was repeated until there was no significant difference in the tissue sample weights (>0.05 g) when compared with the previous freeze-drying cycle. All control and test tissue samples were assessed for moisture content with an IR-200 Moisture Analyzer (Denver Instrument, Denver, CO) and all tissues analyzed had a moisture content of ranging from 12 to 14%. Lyophilized samples were pulverized into a fine powder and the samples were placed in a −80°C freezer until bioassay initiation.

### Bioassay Methodology

Bioassay methodology largely followed the methodology described by Levine et al. (2016). Concentration-dependent responses for *H. zea* growth inhibition with lyophilized tissue from MON 87751, MON 87701, and MON 87751 × MON 87701 × MON 87708 × MON 89788 were characterized in 7-d diet-incorporation bioassays. *Helicoverpa zea* eggs were obtained from Benzon Research (Carlisle, PA). Eggs were placed into covered boxes and held at a target temperature of 10°C prior to incubation for hatching at 27°C. Treatment concentrations were chosen to characterize concentration–response relationships and to accurately estimate median effect values. For treatments with MON 87751, MON 87701, or MON 87751 × MON 87701 × MON 87708 × MON 89788, one replicate for each concentration was tested. Test concentrations ranged from 0.16 to 10 µg tissue/ml diet with a two-fold separation factor between concentrations. Three conventional control replicates, each containing 10 µg tissue/ml diet, were included with each replicate assay. Bioassays in our laboratory have shown that these small microgram amounts of lyophilized soybean tissue incorporated into diet do not have an impact on *H. zea*.

Treatments were prepared by mixing 5 ml of purified water with finely ground lyophilized leaf tissue and then adding 20 ml of warm (52°C) agar-based multispecies diet (Southland Products). Diet was then vortex-mixed until visually homogeneous. A volume of 1.0 ml of diet was aliquoted into individual wells of 128-well bioassay trays (Benzon Research, Carlisle, PA) and the diet was then allowed to cool and solidify. One newly hatched larva (<30 h from the first observed hatching) was placed in each well. Larvae were individually and impartially added to each treatment in a nonsystematic manner. Once one treatment was completed, another treatment was nonsystematically selected, and individual larvae were added to each well. Each replicate contained a target number of 16 *H. zea* that were individually housed and covered with a ventilated adhesive cover. At the end of the bioassay, the total number of insects, the number of surviving insects, and the combined insect weight of the surviving insects were recorded.

### Concentration Response Modeling and Assessment of Additivity

Concentration response modeling and median effect concentrations for growth inhibition (EC_50_) and their associated asymptotic 95% CI were estimated with a 3-parameter logistic equation (Van Ewijk and Hoekstra 1993). The median effect level was selected as the measurement endpoint to compare observed and predicted values because it is the most statistically reliable endpoint to be estimated from a concentration–response curve (Newman 2013). The model was constrained to use a shared parameter for control weight across treatments. In addition, the model was constrained to use a shared slope parameter across treatments because there was not a significant difference in slopes across treatments (*P* > 0.050). No generally accepted procedures exist for statistically significant deviations from additivity; however, many statistical methods and study designs that address biological variability in interaction analysis have been published (Cassee et al. 1998). For this analysis, antagonism (less than additivity) would be concluded if the upper bound of the 95% CI for the observed median effect level was less than the predicted median effect level using the concentration addition model. A GTA interaction would be concluded if the lower bound of the 95% CI for the observed median effect level was greater than the predicted median effect level using the concentration addition model. Additivity would be concluded if the predicted median effect level was captured within the 95% CI of the observed median effect level (Lewis and Perry 1981, Tabashnik 1992, Jonker et al. 2012, Levine et al. 2016) or within the 95% CI bounding the isobole (Borgert et al. 2005). The isobole method is based on concentration addition and is carried out by constructing a graph with the axes of the graph representing doses of the two substances on a linear scale (Loewe and Muischnek 1926, Berenbaum 1985). A line joining the iso-effective doses for the single events predicts the combinations that will yield the same effect, provided the interaction is additive. For this analysis, the iso-effective dose that was selected was the median effect concentration.

For the concentration addition model, predicted EC_50_ values were calculated as follows: 1/predicted EC_50_ = π*a*/EC_50_*a* + π*b*/EC_50_*b*, where π*a* and π*b* are the proportions of the two single trait products, *a* = MON 87751 and *b* = MON 87701 in the combined trait product MON 87751 × MON 87701 × MON 87708 × MON 89788. For MON 87751 and MON 87701, π*a* and π*b* are equal to one because they are both expressed in the combined trait product and expression is comparable for the singles and combined trait product (Finney 1971). Combined responses for MON 87751 and MON 87701 under the response addition model were calculated for each treatment level following the formula for multiplicative responses (Finney 1971): P_stack_ = 1 − (1 − P_MON87751_) (1 − P_MON87701_), where P_stack_ equals the predicted response of the stack based on the responses of the individual events. Predicted and observed concentration responses were fit with the 3-parameter logistic regression to estimate EC_50_ values and their associated asymptotic 95% CI. Predicted and observed EC_50_ values based on responses for the stack were compared as described for the concentration addition model.

## Results and Discussion

Combining PIPs in GE soybeans has become an important strategy to delay potential insect resistance and improve the spectrum of controlled pests. Recently, DAS-8149-2 which produces the Cry1F and Cry1Ac proteins to control lepidopteran pests in soybean was recently commercialized (USEPA 2014, Marques et al. 2018). Monsanto has also recently developed a multiple PIP soybean product to control lepidopteran pests in soybean by combining events MON 87751 and MON 87701 through conventional breeding. This combined trait product was bred with MON 87708 that confers dicamba tolerance and MON 89788 that confers glyphosate tolerance. Many regulatory authorities require interaction studies to evaluate the potential for an interaction between PIPs in stacked products that could affect a human or environmental risk assessment. To support global stack registrations for MON 87751 × MON 87701 × MON 87708 × MON 89788, a PIP toxicological interaction study was conducted using a sensitive insect bioassay. This study tested the null hypothesis of no interaction between the insecticidal activity expressed by MON 87751 and MON 87701.

The design of interaction studies is a subject that has received a lot of attention in the literature and recommendations have been made on how to implement economical study designs (Cassee et al. 1998). The experimental design of interaction studies can vary based on activity and specificity of the substances as well as the organism tested and endpoint measured. The design used in this study for assessing potential interactions is the fixed-ratio design or ray design. With this design, the concentration–response for each substance was characterized along with the concentration–response for the combination as expressed in the stack. The fixed-ratio design also provided a good visual interpretation of the results and lends itself to an easy analysis of deviations from additivity. An assessment of a potential interaction for fixed-ratio design studies is commonly based on constructing a 95% confidence interval (CI) around the fitted effect of the response and then analyzing whether the predicted effect is captured by the CI (Tabashnik 1992, Jonker et al. 2012, Levine et al. 2016). The advantage of this design and approach is that it considers the uncertainty in the prediction.

**Fig. 1.**
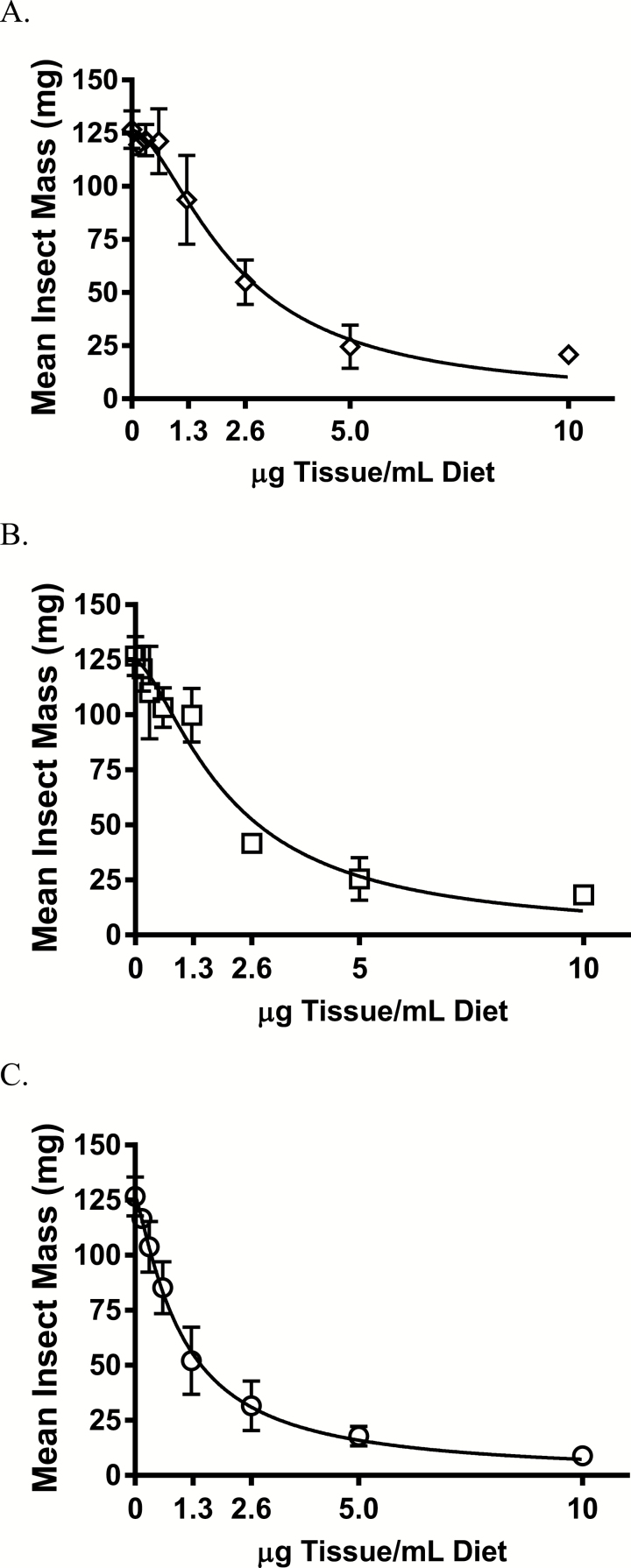
Concentration responses for corn earworm (Lepidoptera: Noctuidae, *Helicoverpa zea*) growth inhibition after 7-d feeding on leaf tissue derived from (A) MON 87751, (B) MON 88701, and (C) MON 87751 × MON 87701 × MON 87708 × MON 89788 incorporated into an artificial diet. Addition of EC_50_ values for MON 87751 and MON 88701 were consistent with predicted EC_50_ values using the concentration addition model and concentration responses for MON 87751 × MON 87701 × MON 87708 × MON 89788 were comparable.

*Helicoverpa zea* demonstrated concentration-dependent growth inhibition after 7 d of feeding on leaf tissue derived from MON 87751, MON 87701, and MON 87751 × MON 87701 × MON 87708 × MON 89788 incorporated into an artificial diet (Fig. 1). Mortality in the control and treatment groups was low with no single replicate exceeding 6 and 20%, respectively. EC_50_ values were estimated to be 1.6 µg tissue/ml diet and 1.9 µg tissue/ml for MON 87751 and MON 87701, respectively (Table 2). The observed EC_50_ value for the stack was 0.86 µg tissue/ml diet (Table 2), with the stacked product demonstrating an approximate two-fold increase in activity over the single events. A formal assessment of additivity for MON 87751 and MON 87701 in the stack was performed with the concentration and response addition models. Under the concentration addition model, the predicted EC_50_ value for the stack of 0.88 µg tissue/ml diet, which is captured within the 95% CI (0.64 to 1.1 µg tissue/ml diet) for the predicted EC_50_ value (Table 2). The difference between the observed and predicted EC_50_ values for the stack was only 2%.

**Table 2.** Estimated 7-d soybean podworm (*Helicoverpa zea*) EC_50_ values and 95% confidence intervals (CI) for MON 87751, MON 87701, and MON 87751 × MON 87701 × MON 87708 × MON 89788 and the predicted EC_50_ value for MON 87751 × MON 87701 × MON 87708 × MON 89788 using the concentration addition model

Treatment	EC_50_ value (µg tissue/ml diet)*	95% CI	Predicted EC_50_ value (µg tissue/ ml diet)^†^	% Deviation for predicted EC_50_ value
MON 87751	1.9	1.4–2.4		
MON 87701	1.6	1.2–2.1		
MON 87751 × MON 87701 × MON 87708 × MON 89788	0.86	0.64–1.1	0.88^†^	2.3%

* Slopes were not significantly different across the treatments (*P* > 0.05); therefore, a shared logistic slope of 1.0 was used for joint concentration response modeling.

^†^ The 95% CI for the predicted EC_50_ value was estimated to be 0.67–1.1 µg tissue/ml using the Delta method.

**Fig. 2.**
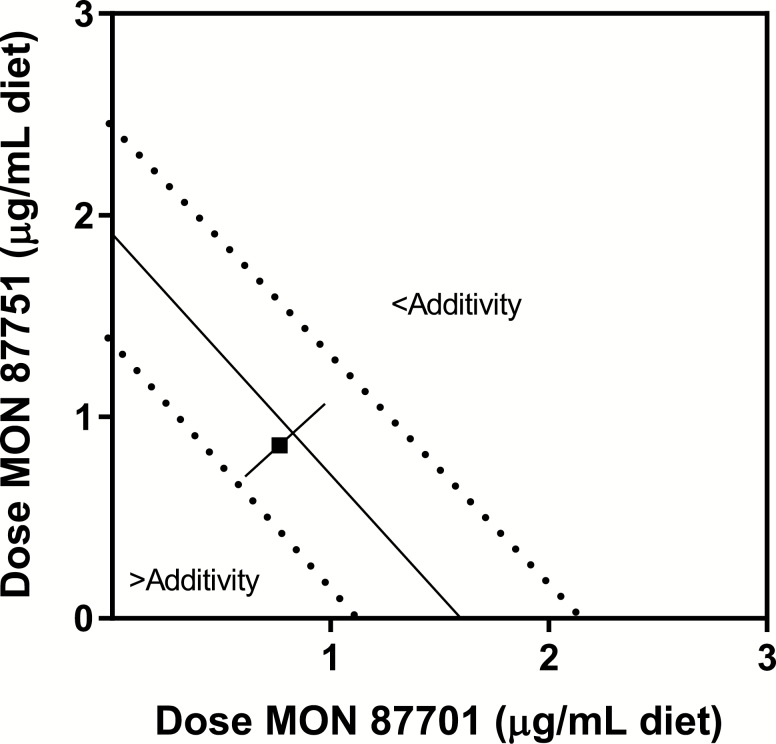
The line joining the EC_50_ values for MON 87751 and MON 88017 shows similar predicted and observed 50% effect levels for the combined activity of MON 87751 and MON 88017 against corn earworm (Lepidoptera: Noctuidae, *Helicoverpa zea*) under the assumption of additivity. The dotted lines represent the 95% confidence interval for the isobole for MON 87751 and MON 88017. The observed activity is represented by a square (■) and the bar indicates the 95% confidence interval.

The small difference between predicted and observed activity has been further illustrated with an isobologram (Fig. 2). An isobologram shows concentration ratios that give constant biological activity and assist in a visual assessment of potential deviations from additive activity. Historically, isobolograms for median “50^th^ centile” effect levels have been used to graphically illustrate potential deviations from the line of additivity. However, a confidence interval bounding the line of additivity is rarely included with isobolograms and these intervals can be used to illustrate the uncertainty around predictions of combined additivity. Borgert et al. (2005) proposed that this interval should be routinely added to isobolograms to help evaluate whether combined activity was consistent with additivity. An isobologram with a 95% CI bounding the line of additivity for the median 50^th^ centiles for MON 87751 and MON 87701 was included to provide a visual assessment of potential deviations from additivity. The isobologram shows that the observed EC_50_ value is well within the confidence intervals of the EC_50_ values for the individual events. Each of these comparisons, based on the concentration addition model, demonstrates no interaction (i.e., additivity) between the PIPs produced by MON 87751 and MON 87701.

The predicted activity for the stack with the response addition model is highly comparable with the predicted activity for the stack with the concentration addition model (Tables 2 and 3). Concentration–responses for each single and observed and predicted responses for the stack are illustrated in Supp Fig. 1 (online only). The predicted EC_50_ value under the response addition model of 1.1 µg tissue/ml diet is captured within the 95% CI for the observed EC_50_ value for the stack (0.79 to 1.4 µg tissue/ml diet), demonstrating that the combined response to MON 87751 and MON 87701 is consistent with additivity (Table 3). Comparable predictions for median effect levels from both additivity models is not unexpected and a slightly more conservative prediction of combined activity under the concentration addition model is also not unexpected since this model commonly provides more conservative predictions (Finney 1971). Nonetheless, both models support the hypothesis of no interaction. Since both models differed by less than 10% between predicted and observed median effect values, either approach was acceptable for assessing deviations from additivity for the stack.

**Table 3.** Estimated 7-d soybean podworm (*Helicoverpa zea*) EC_50_ values and 95% confidence intervals (CI) for MON 87751, MON 87701, and MON 87751 × MON 87701 × MON 87708 × MON 89788 and the predicted EC_50_ value for MON 87751 × MON 87701 × MON 87708 × MON 89788 using the response addition model

Treatment	EC_50_ value (µg tissue/ml diet)^†,*^	95% CI	Predicted EC_50_ value (µg tissue/ml diet)	% Deviation for predicted EC_50_ value
MON 87751	1.9	1.3–4.4		
MON 87701	1.9	1.6–2.3		
MON 87751 × MON 87701 × MON 87708 × MON 89788	1.1	0.79–1.4	1.2^†^	9%

* Slopes were not significantly different across the treatments (*P* > 0.05); therefore, a shared slope of −1.4 was used for joint concentration response modeling.

^†^The 95% CI for the predicted EC_50_ value was estimated is 0.9–1.6 µg tissue/ml diet.

The conclusion that the combined activity of MON 87751 and MON 87701 on a sensitive insect species is consistent with additivity agrees with several studies that evaluated subcombinations of the *Bt* proteins produced by MON 87751 and MON 87701. Previously, the potential for interaction with Cry1A.105 and Cry2Ab2 proteins was evaluated as part of the registration of MON 89034 maize and activity was shown to be additive with *H. zea* and the European corn borer (Lepidoptera: Crambidae, *Ostrinia nubilalis*) using mortality and development stage as endpoints (USEPA 2009c). Similarly, the combined activity of Cry1Ac and Cry2Ab2 protein has been shown to be additive with *H. zea* (Greenplate et al. 2003). Furthermore, Luo et al. (2007) showed with reciprocal binding tests with brush border membrane vesicles from *Helicoverpa armigera* (Lepidoptera: Noctuidae) that Cry2Ab could not displace Cry1Ac. Hernandez-Rodriguez et al. (2008) confirmed the results of these earlier studies with two Heliothine species by demonstrating that Cry2Ab does not compete for binding sites with Cry1Ac. In contrast to these competition binding studies, Ibargutxi et al. (2008) reported a 3-fold lower median effect concentration for the combined activity of Cry1Ac and Cry2Ab with *Helicoverpa armigera* but only at a Cry1Ac:Cry2Ab2 ratio of 1:4 but did not observe this difference at the other tested ratios of 1:1 and 4:1. No mechanistic information was provided by Ibargutxi et al. (2008) to understand the basis for the reported difference in predicted and observed activity. This result is inconsistent with Luo et al. (2007) and Hernandez-Rodriguez et al. (2008), which as previously discussed, showed with reciprocal binding tests with brush border membrane vesicles from *Helicoverpa armigera* that Cry2Ab could not compete Cry1Ac. Furthermore, high heterogeneity in larval weights was observed for Cry2Ab2 in Ibargutxi et al. (2008), which could have confounded the assay. It was also not clear from the paper whether assays with the individual proteins and the different combinations were tested in in head-to-head assays. This is important because potency estimates (i.e., LC_50_ values) are not biological constants (Casarett et al. 2003). Recently, Belden and Brain (2018) concluded that difference between predicted and observed values that are less than five-fold are not indicative of a GTA interaction and this five-fold criterion is consistent with guidance from the European Food Protection Agency (EFSA) that considers a less than five-fold difference not indicative of a significant interaction (EFSA 2013). This five-fold criterion was re-emphasized in EFSA’s recent draft guidance on mixtures testing and assessment for microbial and biological pesticides (EFSA 2018). As previously mentioned, the USEPA considers differences between predicted and observed values of <10-fold not be impactful to a risk assessment for the combined trait product (USEPA 2009a).

It was argued by De Schrijver et al. (2015) that potential interactions between combinations of *Bt* proteins with overlapping specificity are difficult to predict. However, it has been well established in the scientific literature that GTA interactions are exceptionally rare (Syberg et al. 2009, NRC 2013, Cedergreen 2014). In EPA’s review of *Bt* Cry protein interaction studies, that have been submitted to support combined trait insecticidal products, no combinations of Cry proteins demonstrated significant GTA interactions (USEPA 2009a). In 2012, a committee formed under the National Academy of Sciences to address uncertainties in the assessment of pesticide mixtures recommended that regulatory agencies only consider GTA interactions quantitatively when the best available scientific evidence supports the evaluation (NRC 2013). In other words, in the absence of any data that would support the hypothesis for a GTA interaction, the effects analysis should proceed under the assumption of no-interaction. Therefore, interaction studies for PIPs are generally viewed by the USEPA as data that inform a bridging assessment from the single events to stacked products.

Previously, the environmental safety of MON 87751 and MON 87701 has been evaluated (USEPA 2010, USEPA 2015a). For MON 87751 and MON 87701, it was concluded that adverse effects to NTOs, including birds, wild mammals, freshwater and marine/estuarine fish and invertebrates, nontarget insects, honey bees, soil invertebrates, and terrestrial and aquatic plants, are not anticipated. This assessment was primarily based on results from laboratory studies where large margins of exposure were established between no effect levels for nontarget organisms and predicted environmental exposure levels (USEPA 2010, USEPA 2015a). Based on these results from studies on NTOs, and information on habitat requirements for threatened and endangered, the USEPA made a “no effect” determination for direct and indirect effects to threatened and endangered species and their habitats from cultivation of MON 87751 and MON 87701 and by extension a combined trait product with MON 87751 and MON 87701.

### Conclusions

The results from bioassays with *H. zea* demonstrate that the combined activity of MON 87751 and MON 87701 is consistent with additivity. Overall, there was good agreement with predicted and observed median effect levels for the stack under both the concentration and response addition models. It is generally understood that exposed organisms are not susceptible to combined effects when exposure concentrations for components with different mechanisms of action do not exceed their no effect levels (Price 2010, Kortenkamp and Altenburger 2012). The NTO assessments for MON 87751 and MON 87701 were based on no observed effects levels (NOELs) and there were sufficient margins of safety (i.e., ≥10-fold) between the NOELs and field exposure levels making the chance of additive effects at field-relevant exposures unlikely. These margins of safety reflect the high taxonomic specificity of the *Bt* proteins produced by MON 87751 and MON 87701 for lepidopterans (Widner and Whiteley 1989, Van Rie et al. 1990). Therefore, a finding of no interaction, and no unreasonable adverse effects to NTOs at exposure levels that exceed field exposure levels, provides a mechanism to bridge back to the independent assessments for the individual products that demonstrate the environmental safety of MON 87751 and MON 87701 (USEPA 2009a, Levine et al. 2016).

## Supplementary Material

nvz082_suppl_Supplementary-Material
