# Corrigendum to: Assessing the Potential for Interaction in Insecticidal Activity Between MON 87751 × MON 87701 Produced by Conventional Breeding

**DOI:** 10.1093/ee/nvz108

**Published:** 2019-09-26

Correction of “Steven, L. L., M.F. Jennifer, and P.U. Johua. 2019. Assessing the Potential for Interaction in Insecticidal Activity Between MON 87751 × MON 87701 Produced by Conventional Breeding. Environ. Entomol.”

Doi: 10.1093/ee/nvz082

This article incorrectly displayed an author name online. The author’s correct name is Joshua P. Uffman. This has been corrected online and in print. The authors regret this error.

