# Supplementary material for: Assessing the Potential for Interaction in Insecticidal Activity Between MON 87751 × MON 87701 Produced by Conventional Breeding
Source: Environ Entomol. 2019 Jul 1;48(5):1241–8. doi: 10.1093/ee/nvz082 (PMC6766477; doi:10.1093/ee/nvz082)
Supplement: nvz082_suppl_Supplementary-Material [file nvz082_suppl_supplementary-material.docx]

| **Supplementary Figure 1.** Concentration-responses for soybean podworm (SPW, Helicoverpa zea) growth inhibition for (a) MON 87751, (B) MON 88701 and (C) the stack MON 87751 × MON 87701 × MON 87708 × MON 89788. Predicted and observed EC50 values for the stack using the response addition model were comparable as shown by the overlap of the dotted 95% confidence intervals for each response.  **Supplementary Figure 1** |
| --- |
| A. |
|  |
| B. |
|  |
| C. |
|  |
